# “The kind of support that matters to exclusive breastfeeding” a qualitative study

**DOI:** 10.1186/s12884-021-03590-2

**Published:** 2021-02-09

**Authors:** Dasheka Zukiswa Theodorah, Rala Ntombana Mc’Deline

**Affiliations:** Department of Nursing Science, Faculty of Health Sciences, University of Fort Hare, 50 Church Street, Eastern Cape East London, South Africa

**Keywords:** First‐time mother, Support, Exclusive breastfeeding, Resilience

## Abstract

**Background:**

Worldwide, only 41 % of infants are exclusively breastfed for the first six months while South Africa has an alarming figure of only 12 %. First-time mothers are inexperienced in the initiation and maintenance of exclusive breastfeeding, hence a need for support. Data on forms and quality of exclusive breastfeeding support as experienced by first-time mothers is minimal. The study explored the exclusive breastfeeding support available to first-time mothers in the Buffalo City Metro, South Africa.

**Methods:**

A qualitative explorative, descriptive and contextual study, and a non-probability, purposive sampling was used with 10 first-time mothers within the first six months postpartum. The in-depth face-to-face semi-structured individual interviews for data collection and Creswell’s steps of thematic analysis were used.

**Results:**

Two themes emerged; challenges, empowerment, support and resilience during initiation of exclusive breastfeeding, and diverse support and resilience during maintenance of exclusive breastfeeding. First-time mothers received practical support majorly from nurses and other mothers during the initiation; social support was from family members, friends, and community members for the maintenance of exclusive breastfeeding. Sometimes there was a disjuncture between practical support from nurses and that from family members and the community. There were instances where the support was needed but not given or not supportive of exclusive breastfeeding.

**Conclusions:**

These findings illustrate that professional, practical and social support for first-time mothers is crucial in the initiation and maintenance of exclusive breastfeeding for the first six months. Timing and the kind of support given to these mothers is crucial for successful exclusive breastfeeding.

## Background

First-time mothers need all kinds of support to succeed in Exclusive Breastfeeding (EBF). Inappropriate feeding practices are common for first-time mothers due to several reasons. Pressure from family members and the community may cause the first-time mother to choose a feeding method she may not be able to sustain [1, 2]. Also, mixed messages from health care personnel result in inappropriate feeding practices [3, 4]. First-time mother’s breastfeeding support needs are described well through House theoretical framework [5]. The framework emphasized four supportive behaviour categories, namely, informational, instrumental, emotional and appraisal. The informational support conveys behaviours such as guidance, counsel, and information, while instrumental behaviour focuses on the practical skills which the first-time mother can learn and improve the breastfeeding practice [5]. Furthermore, emotional behaviour is concerned with issues around trust, empathy and caring behaviour, while appraisal focus on positive feedback, encouragement and motivating the first-time mother to continue breastfeeding despite challenges. Supportive interventions increase the number of women who exclusively breastfeed for the first six months successfully [6]. Failure to provide appropriate EBF support to first-time mothers can lead to inadequate infant feeding practices.

Worldwide there are inadequate infant feeding practices, varying amongst the regions [7]. Furthermore, suboptimal EBF remains the key contributor to the leading causes of child morbidities, namely, diarrhoea and acute respiratory infections. An infant who is not exclusively breastfed for the first six months of life has a fourteen times likelihood of dying of all causes, especially, diarrhoea and pneumonia compared to an exclusively breastfed infant. Worldwide, only 43 % of newborns initiate breastfeeding while only 41 % of infants are exclusively breastfed for the first six months of life [8]. In South Africa, including the Eastern Cape Province, of which Buffalo City Metropolitan (BCM) is part of, only 12 % of infants are exclusively breastfed for six months despite the 90 % initiation rate [9]. To mitigate this challenge and improve the low exclusive breastfeeding for six months rates, there is a need to support first-time mothers from the antenatal care period onwards [10].

During antenatal care, nurses support first-time mothers through information about breastfeeding practice, and during labour, practical support is given by assisting mothers to initiate breastfeeding [10]. All mothers and babies without complications after childbirth are discharged home within six hours of the postnatal period. Once discharged, first-time mothers majorly depend on the family members, friends, and community for continued support on EBF. The support significantly influences the practice and success of EBF [11]. Furthermore, the social, cultural, economic, biological, psychological aspects, and health professionals’ support are critical in the maintenance of EBF [12]. Therefore, there are various aspects of support necessary to ensure the maintenance of EBF. A gap remains on the aspects of support that may be effective in the maintenance of EBF by first-time mothers in BCM.

Considering the BCM Health District context, increase in infant mortality rate and under five years related diarrhoeal diseases, and possibly owing to the infant feeding challenges, it was thus vital to describe the support available to first-time mothers to exclusively breastfeed for at least six months, in BCM, South Africa.

## Methods

A qualitative approach, with an exploratory, descriptive and contextual research design was most appropriate to understand the first-time mothers’ available EBF support within their natural environment [13, 14]. The descriptions were based on the constructivist inquiry of multiple realities which can only be understood according to the perspective of the individuals who have experienced the phenomenon, which is first-time mother’s available support [15]. First-time mothers were the population for this study while the target population was first-time mothers who had given birth to a live, full-term and healthy infants, regardless of the birthing mode in one of the Community Health Centres (CHCs) or hospitals within BCM, South Africa. BCM is an urban area comprising majorly low socio-economy households, both formal, informal settlements and Rural Development Plan (RDP) houses. The result is a high population density of 300 persons per square kilometre because of rapid urbanisation with an average household size of 3.3 [16]. This setting impacts the kind of support available for first-time mothers, as these mothers are inexperienced in EBF.

Once the University Research Ethics Committee granted ethical approval and permission recognized by the BCM sub-district health, informed consent was sort from participants before data collection [14].

First-time mothers were recruited during the postnatal visits via poster advertisements displayed in both English and Xhosa in the health institutions with researchers’ contact details. Information sessions to outline the study objectives were held in the health institutions. Interested participants sent a “please call’ while others preferred to call for clarity. Interested participants gave verbal consent and a date, time and place for interviews were then set, where the written consent was signed. A non-probability, purposive sample of 10 first-time mothers was used [17]. In-depth individual face-to-face interviews using a semi-structured interview guide were conducted at the participant’s homes or the local clinic according to participants’ choice. Pseudo names were assigned to all participants using numbers to ensure confidentiality ‘Participant 1’, 2, 3, etc.). These are the questions asked from participants; what assisted or stopped you from maintaining breastfeeding? Are there people who supported you/ encouraged you to continue exclusive breastfeeding? Who supported you the most? How did he/she support you? What type of support did you expect and from whom? Finally, what ways can be used to encourage first-time mothers to exclusively breastfeed? The principle of data saturation was useful to consider the extent to which additional data is likely to change the finding and decide the sample size. Data saturation was achieved by the eighth interview and two more interviews were done in which no new findings were being revealed. Interviews were audio-recorded and transcribed verbatim. Data were then analysed according to Creswell’s steps of thematic analysis [17]. The researcher and the co-coder independently read the transcriptions several times and coded the data line-by-line manually. After coding, through a consensus process; categories, sub-categories and themes were formulated [18]. During these discussions, the researcher and the co- coder considered if a theme or subtheme represented the views of all participants, and rich thick descriptions were used.

Trustworthiness was ensured by observing the principles of transferability, confirmability, dependability, and credibility [19, 20]. Information on the culture, the context, and characteristics of first-time mothers from the BCM were provided to allow transferability depended on the extent to which the findings can be transferred to other settings or groups based on the degree of similarity to BCM. After data transcription and translation to English, an independent translator was used to back translate to the original language of the interview to check for accuracy for confirmability. The researcher analysed and coded data from the transcript and waited at least a day or two to re-code the same data and compared the results before combining two and more transcripts to ensure dependability. The credibility of the data was enhanced by having two researchers independently code and interpret the data, providing a basis for reflective discussions.

## Results

Two themes emerged from the data analysis. These were; (1) “Challenges, empowerment, support and resilience during initiation of exclusive breastfeeding” with a sub-category, “Support and resilience during EBF initiation”, and (2) “Diverse support and resilience during maintenance of exclusive breastfeeding” with a sub-category, “Support and resilience during EBF maintenance”.

### Support and resilience during EBF initiation

Some first-time mothers reported having received support during the antenatal period and the initiation of exclusive breastfeeding.

*“I told myself during pregnancy that I am going to breastfeed, as a breastfed baby is healthy*.” (P9).

“*At the clinic, we are taught to exclusively breastfeed the baby and then give food and other things after six months.“ (P1)*.

Most first-time mothers emphasized that the baby was brought to them by nurses and were asked to start breastfeeding.

“*I was given the baby; it was not that long. I kept him with me…”* (P1).

*“I was told to wait a few minutes, 5 minutes, I waited. My baby was brought in. Nurses said I must pull the nipple and put it in his mouth” (P6)*.

*“The Nurse gave me the baby and told me to sit up and breastfeed” (P2)*.

While participant one reported that her baby was brought to her within a reasonable time, she did not feel supported according to the kind of support she needed.

*“I was never shown that you do this way for you to breastfeed. … the nurse was busy with other things but she was saying put the breast in. I expected that when I get the baby I will be shown; how the baby sucks, hold the baby this way, insert the nipple this way …” (P1)*.

While this mother was not happy with the kind of support she initially received, some received a demonstration on positioning and attachment of the baby to the breast and saw it as supportive.

*“I was not even able to hold him, how am I going to hold him. The nurse showed me how to do it. Even when breastfeeding what to do,” (P3)*.

*“I was taught how to breastfeed and the way of holding the breast for him. I was taught by the nurse that delivered me.” (P4)*.

*“They showed me that ‘hold him this way’.” (P5)*.

*“The nurses showed me how I need to position him here on my breast” (P10)*.

*“The nurse … showed me that I need to hold him with my left arm and I supported with my right one…” (P2)*.

Practical support to manage early breastfeeding challenges such as ‘not enough milk’ through the supply of tablets and giving of injection were seen as supportive.

*“Nothing was coming out. I was given tablets. ….. then it started to come now” (P7)*.

The support was from mixed sources; information and practical support were from nurses and social support from family members, friends, and community members. Other mothers who delivered around the same time as first-time mothers gave social support by encouraging these mothers by an example.

*“Also seeing from other mothers when they are breastfeeding” (P8)*.

One participant associated her insufficient milk supply with the failure of the nurse to give her practical assistance during the EBF initiation period leading to her eventually mix feeding despite her intentions to EBF.

*“I think that if they have shown me and kept it from the beginning, it was going to be continuous throughout. For an example, I ended up with dry breasts until day three.”* (P1).

These findings emphasize the need for resilience on the part of first-time mothers while learning the skills to EBF and dealing with related challenges including continued support from nurses, to succeed in EBF maintenance.

### Support and resilience during EBF maintenance

During EBF maintenance, the source of social support was mostly from family members, friends, and other community members while information and practical support, which included teaching and observing the first-time mother’s breastfeeding techniques, were from nurses. Scheduled postnatal follow-up visits within three to six days of delivery were cited as helping support first-time mothers’ maintenance of EBF through practical assistance with breastfeeding challenges and appraisal. One mother who initially did not feel well supported to initiate EBF confirmed that she did get the knowledge and skill later during this period.

*“When I went back to the clinic, that is where I was taught that you breastfeed the baby, how you hold him when you are breastfeeding.*” (P1).

“*Support from the clinic is really good*”. (P3)

“*Yes, there were students that were teaching there. The nurses there at the clinic are supporting me. …they motivate me to breastfeed the baby*” (P9).

Most of the first-time mothers enjoyed the support from the family members, friends, and other mothers in the birthing unit whom they considered more experienced in breastfeeding than they were.

“*Another lady I was with there said it is not the first time she is having a baby. I must request tablets to produce milk. She said ‘I have seven children but my children, I do not have a child that I say he is not breastfeeding’*.” (P7).

“*It is my grandmother and my older sister, my family, and my cousins. When I have a problem, I tell them*.” (P6).

One participant who previously did not have enough milk reported,

“*When I started having tea then I had a lot of milk. My sister said, ‘my sister, I did not breastfeed my child because I was working, you are not working so for your baby to grow quick you need to give the breast’*.” (P7).

Encouragement by nurses seemed to motivate first-time mothers to continue EBF.

*“His weight was already increased; the nurses said ‘well done. I love what you have done’*.” (P5).

While most first-time mothers received support from family members, some explained the pressure received from family and community members to feed formula, porridge or mix-feed, which sometimes they did not appreciate. First-time mothers highlighted socio-cultural aspects of breastfeeding through the comments of family members, friends, and community members’ involvement in the breastfeeding practice. Some comments were, neither accepted nor appreciated by these mothers.

“*Other mothers that normally come to my house to visit my mother say I am keeping the baby hungry. I am supposed to be feeding him solids now.*” (P4).

“*He was crying … my cousins, my friends came to see the baby and they already have children, said ‘I did not breastfeed … I bought a formula … buy him formula milk.”* (P3).

*“I did not want anyone who is going to say, ‘No feed, steal a spoon for him or two spoons of porridge. Yes, there are such like ones, who say, ‘no this child is not getting enough from the breast*.” (P7).

*“I do not feel well because my friends want me to be able to leave the baby behind to go out with them. So, I do not feel well.”* (P9).

One mother highlighted the importance of a supportive relationship between first-time mothers and nurses.

“*It is important that there is a relationship between her and her nurses”* (P1).

She further reported in-depth the need for formulation of first-time mother’s EBF support groups. She highlighted how it should be run to ensure compliance by first-time mothers, including its benefits.

*“It would be beneficial, something nice the availability of those support groups … if the clinic is organising it at least it would give some weight. Maybe it can be done once a month or once a week or fortnightly but there must be interaction among first-time mothers so that they can share their experiences. Soon after breastfeeding the baby, she must attend those things those meetings.”* (P1).

There is, however, a need to include the communities in information sharing on EBF support to avoid conflict between nurses, communities, and first-time mothers. Some mothers explained the socio-cultural difference in breastfeeding practice between healthcare professionals and elderly community members. This was cited as possibly causing conflict between first-time mothers, community members, and the nurses.

“*My mother said, ‘we did not grow up like this, we fed children roasted this and that*.” (P3).

“…*In our homes, you are shown the thing this way but when you arrive at the clinic, all of that is criticised… At home, you are told to use the second finger next to the thumb and the one next to it, you put the breast in and support with your thumb*.” (P1).

Two mothers voiced the need to take advice with caution, as not all advice is good advice. These mothers explained the need to reject unhelpful advice as the motive might not always be genuine.

“… *your child will never gain weight. Your child will never grow well because you are exclusively breastfeeding. Give him porridge. Do not listen to a friend because a friend sometimes misleads*” (P5).

“*People from the streets will advise the wrong things. Their motive … is that as I am getting the grant on the date for grant pay-outs they want to go with me to the shebeen.*” (P6).

First-time mothers reported feeling pressured to feed formula or porridge to their babies against their own choice. Most mothers did not like these suggestions.

*“He was crying … my cousins, my friends came to see the baby and they already have children, said ‘I did not breastfeed … I bought a formula … buy him formula milk.” P3*.

*“I did not want anyone who is going to say, ‘No feed, steal a spoon for him or two spoons of porridge. Yes, there are such like ones, who say, ‘no this child is not getting enough from the breast.” P7*.

Support received by first-time mothers included socio-cultural values which sometimes was in line with the knowledge and skills received by first-time mothers during antenatal care. First-time mothers were able to accept or reject the support that they deemed harmful to the breastfeeding practice and maintenance of EBF.

## Discussion

This study focused on understanding the EBF support available to first-time mothers in BCM. This was necessary because the first-time mothers are inexperienced in EBF and there are other challenges common to breastfeeding women resulting in few mothers maintaining EBF. First-time mother’s lack of experience and breastfeeding challenges can influence the initiation of breastfeeding. To achieve initiation, support is needed. The support received by first-time mothers in BCM was consistent with the support as conceptualized by House in Grassley’s [5] four supportive behaviour categories. The first-time mothers received information on the importance of EBF for six months, the need to make a choice, breastmilk production and challenges of EBF, during antenatal care and after admission for giving birth.

The information was supported by instrumental or practical support by nurses aimed at ensuring the initiation of breastfeeding. The nurse seemed to expect the first-time mother to know how to put the baby onto the breast, as she continued to tell her to put the baby to the breast without demonstrating to her [21]. While nurses are the main support during the initiation phase [22], that support needs to be non-judgemental for the first-time mother to value the support for application in decision-making [23].

One first-time mother was not happy with the kind of support she initially received, while some received a demonstration on positioning and attachment of the baby to the breast and saw it as supportive. Also, some nurses might not be able to offer practical support needed by first-time mothers to initiate breastfeeding due to ward routine or other reasons, some can provide the kind of support needed. There is thus a need for consistency in providing practical support among nurses. Despite the information received most participants reported challenges of inability to initially attach and position the baby during breastfeeding, not enough breastmilk supply, even though most reported improvement with time. The information received prior needed additional support, practical, emotional and/or appraisal support. This is consistent with the findings that even with the information given during antenatal care, mothers still experience challenges leading to early breastfeeding cessation [4, 9, 24] hence a disjuncture between knowledge and practice of breastfeeding mothers [25]. Lack of such practical support leads to frustration for first-time mothers [26]. It is thus important to provide individualised rather than generalised EBF support, to address the information and practical skills needs of each first-time mother [1].

All mothers who do not experience any problems postnatal are discharged home within 24 hours, consistent with the policy, which was the case with most of the study participants. Mothers are also expected back for a postnatal visit within six days for support relating to any problems experienced including feeding-related problems. It is thus important to ensure that breastfeeding initiation is successful, as family members and the community start to support these mothers. The first-time mothers reported that most family members, friends, and community members supported their EBF practice. This is consistent with the findings that not only nurses can provide support but also partners, friends, and family were key in supporting first-time mothers when encountering breastfeeding challenges [23].

There are contrasting findings on the impact of the intentions to EBF on the maintenance of EBF, some mothers resorted to formula feeding or mix feeding as a feeding method of choice despite the good intention to breastfeed [3, 27]. Contrary to the findings, that early cessation of breastfeeding was associated with no intention to breastfeed [28]. At the time of data collection, of the ten first-time mothers that participated, seven maintained EBF, one was mix-feeding while two switched to formula feeding to avoid mix feeding. The two first-time mothers who switched to formula feeding, worked longer periods consistent with research findings [29, 30]. There is thus a need for additional strategies such as increased paid maternity leave days.

There is also a need for individualised support following all aspects of House theoretical framework for the initiation of EBF to be successful. The nurses, as the main support source for these mothers, need to be vigilant of the special needs for each mother. Successful initiation will lead to the maintenance of EBF. Different aspects of support were cited by first-time mothers as helpful, included are information-sharing, practical support and emotional support received including positive motivation from nurses. The practical support included helping with positioning and attachment; supply of food and fluids including fluids perceived to assist with breastmilk production, such as tea and ginger [31].

In this study, while most first-time mothers received support from family members, one first-time mother explained the pressure to mix-feed she received from community members. Advice from family members especially grandmothers was one of the reasons first-time mothers especially teenagers stop breastfeeding [24]. Also, the need to meet the existential needs, such as stopping suffering caused by pain during breastfeeding or baby’s weight loss associated with insufficient breastmilk supply against social conflicts [32]. Furthermore, the social conflicts through the pressure the society put on mothers, where breastfeeding is portrayed more natural than what it is. Hence a mother that fails to breastfeed might be labelled as a bad mother, generating feelings of failure, while if she succeeds to breastfeed is labelled a good mother. Nurses and society, therefore, need to support mothers to succeed in breastfeeding rather than labelling them as good or bad depending on their feeding method. Breastfeeding is not always easy, especially for first-time mothers. There is thus a need for additional strategies such as support groups, breastfeeding education and awareness to increase EBF maintenance. These support groups can even include virtual support groups to ensure continuous support [33].

Also, socio-cultural issues may cause the support from nurses, family, and community members to contrast leading to confusion and frustration [34, 35]. There is thus a need for nurses and community members to forge unity in supporting first-time mothers to ensure the maintenance of EBF for the first six months of life.

## Limitation

It is important to note various limitations in the study’s methodology. The qualitative approach and the sample size of the study were small, thus limiting generalisation of the findings, however, the findings are similar to other studies done both qualitatively and quantitatively, abroad and nationally, including the Eastern Cape Province where this research was conducted.

## Implications for service providers and policymakers

The nurses are not always readily available to assist first-time mothers to initiate breastfeeding because of ward routines. There is, therefore, a need for peer breastfeeding supporters to supplement nurses in giving practical assistance to first-time mothers before discharge.

Even though mothers receive information during antenatal care, it is not easy for them to apply this knowledge during breastfeeding. Nurses, therefore, need to focus on individualised education based on individual needs and practical support.

Mixed messages from the nurses and family members on EBF practices lead to confusion and frustration for first-time mothers. Nurses, need to unite with community members in providing updated, evidence-based practice on breastfeeding.

There are policies in place on breastfeeding, including infant feeding in the context of HIV. However, there is a need for the formulation of compulsory physical or virtual support groups to provide additional support for first-time mothers.

## Conclusions

First-time mothers do need professional, practical and social support in the form of information, emotional support and encouragement for initiation and maintenance of exclusive breastfeeding for the first six months. However, timing and the kind of support given to these mothers is critical for successful exclusive breastfeeding. Individualised support after the delivery of the baby and postnatal period assist the first-time mothers to put the information received during antenatal care into practical use. There is also a need to ensure that the nurses and community members share the same knowledge and skills, to adequately support the first-time mothers succeed in EBF. Also, the formation of support groups whether in a physical or virtual space would bridge the gap for any additional support needed by these mothers.

## Definition of terms

### Exclusive breastfeeding

 Exclusive breastfeeding means that the infant receives only breast milk, no other liquids or solids are given except for oral rehydration solution, or drops/ syrups of vitamins, minerals or medicines [36].

### Resilience

 Resilience exists when the person uses “mental processes and behaviours in promoting personal assets and protecting self from the potential negative effects of stressors”. [37]

## Data Availability

The interview guide used in this study was developed for this study and has never been used before. The datasets used and/or analysed during the current study are available from the corresponding author on reasonable request.

## Abbreviations

BCM: Buffalo City Metropolitan
CHC: Community Health Centre
EBF: Exclusive Breastfeeding
RDP: Rural Development Plan
